# Primary care sentinel surveillance of influenza-like illness and laboratory detections of respiratory viral infections in Denmark, week 40 2021 to week 39 2023

**DOI:** 10.2807/1560-7917.ES.2025.30.40.2500103

**Published:** 2025-10-09

**Authors:** Amanda Bolt Botnen, Lisbet Krause Knudsen, Julie Grau, Henrik Bang, Jens Nielsen, Jesper Krog, Sofie Midgley, Kristina Træholt Franck, Morten Rasmussen, Uffe Vest Schneider, Hanne-Dorthe Emborg, Lasse S Vestergaard, Ramona Trebbien

**Affiliations:** 1 Department of Virology and Microbiological Preparedness, Statens Serum Institut, Copenhagen, Denmark; 2 Department of Infectious Disease Epidemiology and Prevention, Statens Serum Institut, Copenhagen, Denmark

**Keywords:** Sentinel surveillance, respiratory viruses, influenza, sars-cov-2, respiratory syncytial virus, enterovirus, coronavirus, human metapneumovirus, parainfluenza

## Abstract

**BACKGROUND:**

The Danish respiratory virus sentinel surveillance system has monitored influenza-like illness and influenza virus activity for over 30 years. During the last decade, additional virus groups were added. In 2021, the system was updated to include SARS-CoV-2, collect detailed symptomatic data, and transitioned to year-round surveillance.

**AIM:**

To explore the first two seasons of year-round respiratory virus surveillance and the first symptomatic data collected in the Danish primary care sentinel surveillance system.

**METHODS:**

In 2021/22 and 2022/23 seasons, 156 and 147 participating general practitioners, respectively, reported influenza-like illness consultation rates, collected symptomatic data and swabbed patient volunteers. Swabs were sent to Statens Serum Institut for multiplex PCR analysis, with additional characterisation using high-throughput sequencing or type-specific PCR assays for viruses such as influenza, SARS-CoV-2 and respiratory syncytial virus (RSV).

**RESULTS:**

During the two seasons, 4,391 and 6,034 swabs, respectively, were collected and analysed. Year-round surveillance detected an unusually early wave of RSV during 2022/23. While present in nearly all weeks, SARS-CoV-2 showed waves with increased detection. Year-round surveillance also highlighted consistent patterns, such as continuous presence of entero-/rhinoviruses and endemic coronaviruses, as well as parainfluenza virus appearing after influenza virus. Symptom data showed differences by both sex and virus type, e.g. headaches were more commonly reported by women with RSV.

**CONCLUSIONS:**

Our findings highlight the value of year-round respiratory virus surveillance in identifying both atypical virus activity and consistent patterns outside the winter season. Symptom data suggest the need for further research into sex-specific symptom patterns.

**Key public health message**

**What did you want to address in this study and why?**

Primary care sentinel surveillance of respiratory viruses was usually performed in the winter season, but since the COVID-19 pandemic, Denmark has conducted year-round surveillance. We examined the first two seasons of integrated, year-round surveillance in Denmark, which, for the first time, included an itemised symptom checklist. This allowed us to investigate the symptoms associated with certain virus infections and assess year-round virus activity.

**What have we learnt from this study?**

Our findings demonstrate that while influenza-like illness is more common during winter, many respiratory viruses circulate year-round or can exhibit seasonal waves throughout the year, sometimes earlier or later than expected, such as for RSV or SARS-CoV-2. Data also showed differences in symptoms experienced with viral infection by age and sex.

**What are the implications of your findings for public health?**

Our findings underscore the importance of maintaining year-round respiratory virus surveillance to detect atypical waves and off-season trends, which help explain unexpected spikes in influenza-like illness. Collecting detailed symptom information alongside swab sampling allowed us to examine symptoms associated with specific viruses. However, improvements can be made to digitise the sentinel system to improve data quality.

## Introduction

Influenza and other respiratory viral infections are a major cause of morbidity and mortality every year [1]. Primary care-based sentinel surveillance systems for influenza-like illness (ILI) play a crucial role in tracking the spread and intensity of these viruses, especially during the typical winter seasons, informing public health decision making and planning. This was especially evident during the COVID-19 pandemic, when non-pharmaceutical interventions were implemented to limit the transmission of the severe acute respiratory syndrome coronavirus 2 (SARS-CoV-2), which had a subsequent global effect on the transmission of influenza and other common respiratory viruses [2-6].

The Danish primary care ILI sentinel surveillance system was established in 1994, when Denmark joined the European Influenza Surveillance Network (EISN). At that point, the Danish sentinel surveillance system included ILI activity and laboratory analysis of nasopharyngeal swabs collected from patients in a national sentinel network of participating general practitioners (GPs) during the influenza season running from week 40 to week 20 the following year [7]. The Danish sentinel surveillance system exclusively includes primary healthcare providers. Other nationwide systems for respiratory virus infection exist, such as the project ‘Virus Monitoring in Denmark’, which was a community-based self-testing system [8], the national pathogen-specific surveillance systems using samples from regional clinical microbiological departments, and registry-based surveillance using the 10th revision of the International Classification of Diseases (ICD-10) codes for SARI surveillance [9].

Following the start of the COVID-19 pandemic, the World Health Organization (WHO) and European Centre for Disease Prevention and Control (ECDC) recommended integrating existing national ILI sentinel surveillance systems to include SARS-CoV-2, and to continue surveillance outside of the usual influenza season [10]. Consequently, the sentinel surveillance system in Denmark now represents a comprehensive and integrated approach to monitoring the seasonality of multiple respiratory viruses in primary healthcare [11]. 

Here, we present the setup and results from the integrated sentinel surveillance system in Denmark during the 2021/22 and 2022/23 seasons, which monitors ILI activity, swab laboratory analysis and reported symptoms. To our knowledge, no other respiratory virus sentinel surveillance system currently collects or reports the occurrence of each ILI symptom for the viruses under surveillance.

## Methods

### Study setting

Denmark has a population size of 6 million inhabitants and has approximately 1,700 primary health care practices. Approximately 3,300 general practitioners are employed in primary care, each of which has between 1,100 and 1,600 patients [12] depending on the municipality. 

### System design

The Danish sentinel surveillance system is coordinated by Statens Serum Institut (SSI). Since 1994, primary healthcare providers in the primary sector have been invited to participate through periodic recruitment drives, such as newsletters and articles in professional magazines. Primary healthcare practices can, at any point, sign up to participate in the sentinel surveillance system through SSI’s home page. After signing up, SSI sends swab kits (UTM RT MINI + transport kits with flocked swabs, SSI Diagnostica) to the participating practice. Each practice may have one or several associated GPs. Practices choosing to participate in the sentinel surveillance are termed ‘sentinel practices’ (SPs) and remain SPs until they choose to withdraw.

As of week 40 2021, the Danish sentinel surveillance includes: (i) ILI activity as a percentage of ILI consultations per GP per week, (ii) laboratory analysis of respiratory swab samples for nine virus groups, and (iii) itemised reported symptoms. The surveillance season runs from week 40 up to and including week 39 of the following year. Results for ILI consultations and laboratory analysis are updated weekly on the SSI homepage [13].

### Influenza-like illness consultations

The SPs reported the total number of patients of any age with ILI and the total number of patients seen by participating GPs per week; data were collected per GP. The case definition of ILI, following the ECDC ILI case definition [14], is defined in the Box.

Box **Case definition for influenza-like illness for sentinel surveillance, Denmark, seasons 2021/22 and 2022/23**

(i) Sudden onset of symptoms

AND

(ii) At least one respiratory symptom:

cough, sore throat, shortness of breath

AND

(iii) At least one systemic symptom:

fever >38 °C, myalgia, headache, malaise

Diagnosis of ILI was based exclusively on the patient’s symptoms, without laboratory confirmation of viral infection. The ILI proportion was estimated as a consultation percentage, i.e. the proportion of patients with ILI among the total number of consultations. This ILI proportion was compared with the expected intensity levels as well as a baseline, based on the observed proportion of ILI consultations in the five preceding seasons [15]. Thresholds for intensity are based on the moving epidemic method (MEM).

### Sample collection and laboratory analysis

Each week, GPs took a swab sample from two patients volunteers presenting with ILI. No quotas were applied, and patients of any age or sex could volunteer providing that they exhibited ILI symptoms.

All swabs were sent to the National Influenza Centre for WHO, located at SSI, for laboratory analysis. The Danish sentinel surveillance monitored a range of respiratory viruses: influenza viruses (A(H1N1)pdm09, A(H3N2), B/Victoria and B/Yamagata), respiratory syncytial virus (RSV, types A and B), SARS-CoV-2, endemic coronaviruses (HKU1, NL63, OC43, 229E), human metapneumovirus (hMPV), enterovirus, rhinovirus, parainfluenza virus (types 1–4) and adenovirus. While entero- and rhinoviruses were differentiated by PCR, because of cross-reactivity, these viruses were combined for analytical purposes. 

A multiplex RT-PCR assay was used to detect viruses in respiratory swab samples, combining previously published assays [16-20] with in-house designed assays to cover all the above-mentioned viruses. Subsequent virus culturing, serological and functional assays, and sequencing were used to further characterise some viruses found in submitted swabs following WHO guidelines [21]. 

Interim virus circulation frequencies were defined by the time interval between detections.

### Symptom reporting

For each swabbed patient, the GP filled out a paper-based checklist covering symptom onset and experienced symptoms from the ILI case definition, along with sex, age, height and weight. The symptom checklist covered each symptom described above for ILI, in contrast to checklists during previous seasons, which recorded only whether the patient fulfilled the ILI case definition as a whole. Each swab was therefore paired with an itemised symptom checklist.

## Results

**Figure 1:**
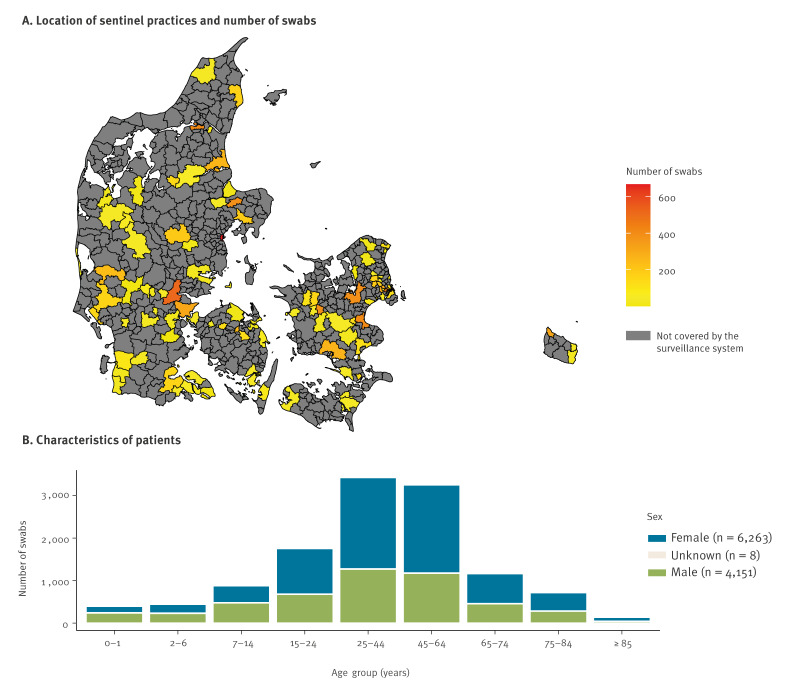
Geographic location and demographic characteristics of patients presenting with influenza-like illness swabbed for sentinel surveillance, Denmark, seasons 2021/22 (n = 156 sentinel practices) and 2022/23 (n = 147 sentinel practices)

The sentinel surveillance had 156 participating SPs in the 2021/22 season and 147 in the 2022/23 season, with 143 SPs participating in both seasons. The SPs were geographically spread across Denmark (Figure 1A). The median number of GPs per SP was two, with a maximum of seven, for both seasons. All age groups were represented, with a majority of swabbed patients being female (6,263/10,422; 60%), except for the youngest age group (0–1-year olds, Figure 1B).

A. The map displays postal codes, colour-coded based on the number of swab samples submitted by sentinel practices to Statens Serum Institut for surveillance during the 2021/22 and 2022/23 seasons (week 40 to week 39 the following year). Approximately 100 of 1,123 postal codes are represented. Areas shown in grey indicate regions not covered by the surveillance system in these seasons.

B. The age and sex distribution of swabs received at Statens Serum Institut, across both seasons.

### Influenza-like illness consultations

**Figure 2:**
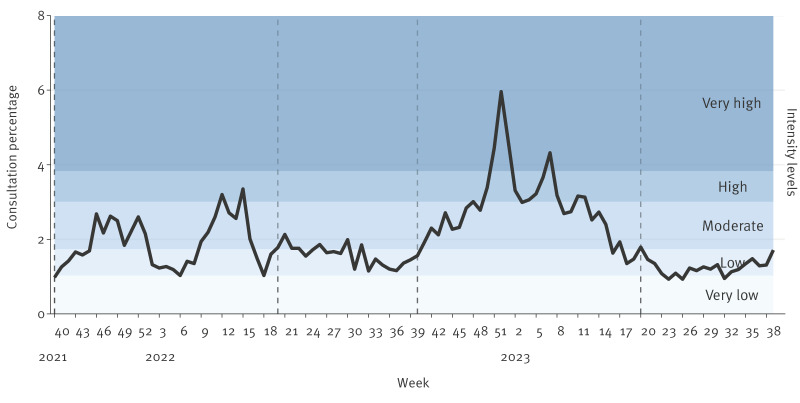
Influenza-like illness consultation percentage in sentinel practices, Denmark, week 40 2021–week 39 2023 (n = 25,145)

In the 2021/22 season, an average of 113 (range: 56–153) SPs per week reported the occurrence of ILI consultations in their clinic, totalling 10,729 consultations. From week 40 2021 until the end of the year, the ILI consultation percentage remained low to moderate (Figure 2). This declined to a low level in the first 8 weeks of 2022. However, from week 9 2022, coinciding with the lifting of all community restrictions at the end of the COVID-19 pandemic, a rapid increase was observed in the share of patients with ILI. According to swab testing from the sentinel system, a steep increase in influenza activity occurred during this time. Influenza activity peaked in week 15 2022 after which the ILI activity declined to a low level.

The black line represents the weekly share of patients with influenza-like illness (ILI) consultations among the participating general practitioners in the sentinel surveillance system from week 40 2021 to week 39 2023. Intensity levels are categorised as: very low (< 1.03%), low (1.03–1.73%), moderate (1.74–3.00%), high (3.01–3.82%), and very high (> 3.83%), based on moving epidemic method (MEM) thresholds [15]. Dashed lines mark the start (week 40) and end (week 20) of the influenza seasons.

In the 2022/23 season, an average of 126 (range: 55–178) SPs reported weekly ILI consultations, totalling 14,416 consultations. From week 40 2022 the share of ILI patients steadily increased and, from week 1 2023, ILI activity repeatedly reached high/very high levels before declining. Laboratory results indicated that this increase was largely because of RSV followed by seasonal influenza virus.

### Laboratory analysis

**Figure 3:**
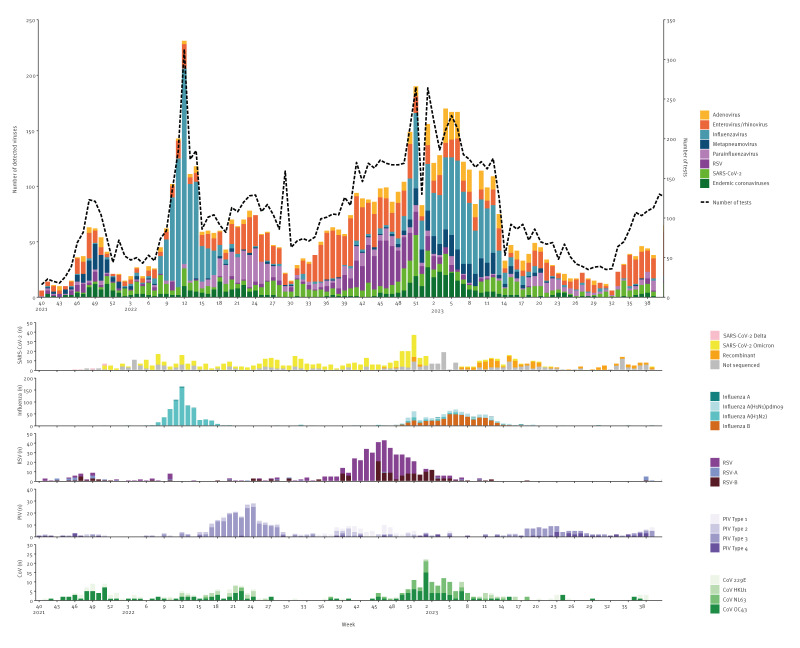
Respiratory virus detections in the sentinel surveillance, Denmark, week 40 2021–week 39 2023

CoV: coronavirus; PIV: parainfluenza virus; RSV: respiratory syncytial virus; SARS-CoV-2: severe acute respiratory syndrome coronavirus 2.

During the 2021/22 and 2022/23 seasons, 4,391 and 6,034 swab samples, respectively, were received at the National Influenza Centre at Statens Serum Institut. In the 2021/22 season, the average weekly positivity percentage was 50% (range: 24–75%), while in the 2022/23 season, the percentage was 51% (range: 19–77%). The number of swab submissions from SPs peaked alongside influenza or SARS-CoV-2 waves. A description of the circulation of each virus during the two seasons is found in Table and Figure 3, and in written in full in the Supplementary Materials.

**Table:** Respiratory virus circulation, Denmark, seasons 2021/22 (n = 4,391 swabs) and 2022/23 (n = 6,034 swabs)

Virus	2021/22				2022/23				
	Detections (n = 2,624)	%	Elevated circulation	Interim frequency	Detections (n = 3,649)	%	Elevated circulation	Interim frequency
Adenovirus	121	2.8	W43–W24	Intermittent	421	7.0	W1–W4	Intermittent
EV/RV	708	16.2	W10–W39	Weekly	786	13.1	W40–W14, W33–W39	Weekly
Influenza virus	729	16.6	W8–W19	Intermittent	884	14.8	W49–W19	Intermittent
HMPV	138	3.2	W41–W07	Intermittent	260	4.3	W48–W22	Rare
Parainfluenza virus	324	7.4	W15–W29	Intermittent	235	3.9	W18–W29	Weekly
RSV	122	2.8	W45–W50	Intermittent	395	6.6	W36–W3	Rare
SARS-CoV-2	301	6.9	W24–W37	Weekly	412	6.9	W40–W5	Weekly
CoV	181	4.1	W41–W27	Weekly	256	4.3	W44–W16	Weekly

CoV: coronavirus; EV: enterovirus; HMPV: human metapneumovirus; RV: rhinovirus; RSV: respiratory syncytial virus; SARS-CoV-2: severe acute respiratory syndrome coronavirus 2.

A season is defined as from week 40 to week 39 of the following year. Number of laboratory-confirmed detections of respiratory viruses and the percentage (%) of the total number of samples per season are provided (n = 4,391 for 2021/22 and n = 6,034 for 2022/23). Elevated circulation refers to the weeks where there were increased detections. Interim frequencies are defined as: weekly = detected every week; intermittent = 2–4 weeks between detections; rare =  > 4 weeks between detections.

The top panel presents the weekly number of laboratory-confirmed respiratory viruses detected through the Danish sentinel surveillance system. All detected viruses, including co-detections, are represented. The black line shows the total number of swabs collected each week. The lower panels further differentiate virus groups, using multiplex RT-PCR assays or sequencing, including SARS-CoV-2 variants, influenza virus subtypes, RSV, PIV, and endemic CoV.

Co-detections with more than one respiratory virus occurred, particularly in children below 2 years of age. Up to four respiratory viruses were detected in a single sample. In contrast, older patients typically only had one respiratory virus detected and rarely two viruses. A bar chart of the number of viruses per age group is provided in Supplementary Figure S1.

**Figure 4:**
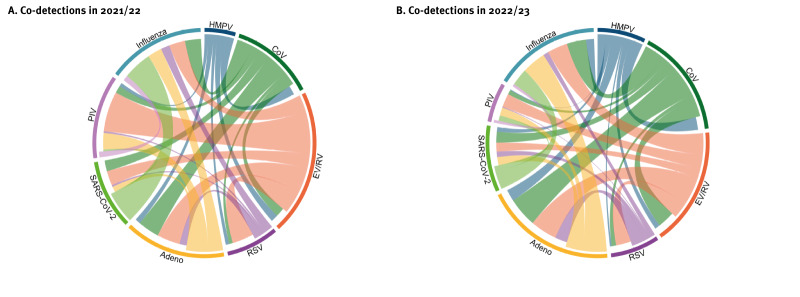
Co-detection pairs of respiratory viruses, Denmark, 2021/22 (n = 169) and 2022/23 (n = 341)

In 2021/22, co-detections were found in 169 samples. Of the 121 swabs with detected adenovirus, 35 (29%) contained one or more of the other viruses. Similarly, 28 (23%) of the 122 swabs positive for RSV also contained other viruses. For other virus groups, the detection of virus pairs was as follows: endemic coronaviruses (37/181; 20%), entero-/rhinovirus (127/708; 18%), parainfluenza virus (42/324; 13%), metapneumovirus (18/138; 13%), SARS-CoV-2 (33/301; 11%), and influenza virus (51/729; 7%). The most common virus pairs were parainfluenza and entero-/rhinovirus (13% of all co-detections), followed by adenovirus and entero-/rhinovirus (8%), and adenovirus and endemic coronaviruses (7%) (Figure 4A).

Adeno: adenovirus; CoV: endemic coronaviruses; EV/RV: entero-/rhinoviruses; HMPV: human metapneumovirus; PIV: parainfluenza virus; RSV: respiratory syncytial virus; SARS-CoV-2: severe acute respiratory syndrome coronavirus 2.

Each chord represents a pairwise relationship between two viruses, with the width of the chord proportional to the magnitude of that relationship, i.e. the number of cases of co-detections.

In 2022/23, 341 patient samples with more than one virus were identified. Co-detections were as follows: endemic coronaviruses (70/250; 28%), adenovirus (101/421; 24%), entero-/rhinovirus (118/786; 15%), parainfluenza (35/235; 15%), metapneumovirus (36/260; 14%), RSV (51/395; 13%), SARS-CoV-2 (54/412; 13%), and influenza (88/884; 10%). The most common co-detections were of adenovirus and endemic coronaviruses (10% of all co-detections), adenovirus and entero-/rhinovirus (9%), followed by adenovirus and influenza (8%) (Figure 4B).

The virus distribution for each age group showed some distinct patterns across both seasons (provided in Supplementary Figure S2). SARS-CoV-2 was more commonly found in the older population, accounting for 28% of detected viruses in the ≥ 85 years age group. In older children and younger adults (< 25 years), influenza virus comprised 39–46% of detected viruses across both seasons, although during the 2021/22 season, influenza virus dominated in the age group ≥ 85 years (33%), likely due to the shift in subtype between the seasons (provided in Supplementary Figure S3). Adenovirus was primarily found in the youngest age groups (0–1 year: 19% and 2–6 years: 23%).

### Symptom reporting

The only non-optional inclusion symptom, sudden onset, was reported as a symptom for 7,901 (75.8%) of all patients regardless of test result. Similarly, 7,046 (71.0%) of included patients reported symptoms fulfilling the ILI case definition. No symptoms were reported in 417 (4.0%) of cases. Of those not fulfilling the ILI criteria, 1,570 (51.9%) were found positive for one or more respiratory viruses. A similar proportion of women and men reported ILI symptoms (4,472 (71.4%) and 2,929 (70.4%), respectively).

**Figure 5:**
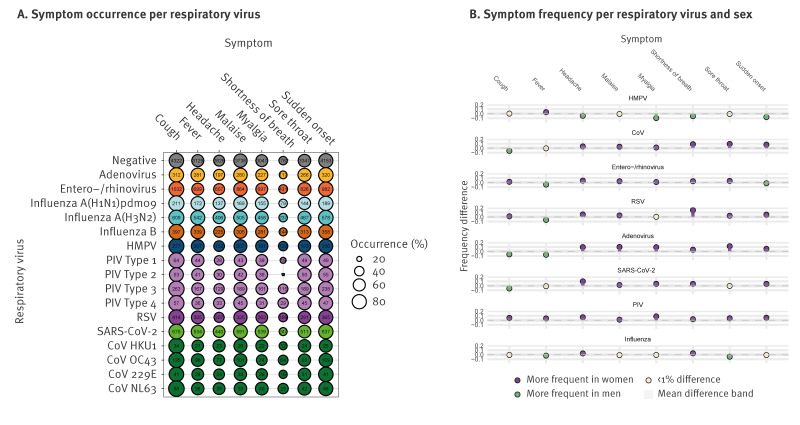
Symptom presentation of respiratory viruses detected in sentinel surveillance, Denmark, week 40 2021–week 39 2023 (n = 4,832)

CoV: endemic coronaviruses; HMPV: human metapneumovirus; PIV: parainfluenza virus; RSV: respiratory syncytial virus; SARS-CoV-2: severe acute respiratory syndrome coronavirus 2.

The most frequently reported symptoms for patients with a single detected virus and at least one reported symptom (n = 4,832) were cough, malaise and sudden onset (Figure 5A). Among the endemic coronaviruses, sudden onset and cough were slightly more common with NL63 and OC43 than with 229E and HKU1. Symptoms reported for parainfluenza virus also showed differences between the four virus types such as shortness of breath, which was less common for types 1 and 2. The influenza virus subtypes had similar symptom presentations.

A. The respiratory virus-associated symptom presentation for patients infected with a single respiratory virus and reporting at least one symptom. Bubble size indicates the occurrence (%) of each virus–symptom combination within the respective virus category. Numbers inside the bubbles show the number of patients exhibiting the symptom. Negative (grey) indicates a negative PCR result. The n for 2021/22 = 1,985 and n for 2022/23 = 2,847.

B. The sex-differentiated frequency difference in reported symptoms for patients with single virus infections and reporting at least one symptom. The mean difference band highlights the overall mean frequency difference for each sex (F: + 0.0536, M: −0.0397).

Men and women reported symptoms differently (Figure 5B). Women more often reported shortness of breath and headaches when infected with a respiratory virus, while men more frequently reported fever. Shortness of breath was reported 15% more frequently in women infected with RSV, and 13% more frequently in women infected with endemic coronaviruses, compared with men. Men reported experiencing fever 8% more frequently than women when infected with adenovirus. Remarkably, during parainfluenza virus infection, women reported all symptoms more frequently than men, while influenza virus infection had similar symptom presentations in both sexes.

## Discussion

Until week 40 2021, the Danish sentinel surveillance operated only during the designated influenza season (weeks 40 to 20). It has since become a year-round, integrated system with three aspects: (i) ILI consultation incidence, (ii) laboratory detection of respiratory viruses and (iii) itemised symptom reporting. This adaptation required minimal changes to the system, in line with the experiences of other European countries such as Sweden and the Netherlands [22].

Peaks in ILI consultation percentages coincided with peaks of laboratory-confirmed respiratory virus activity. The largest peak was seen during the period of elevated influenza circulation in winter 2022/23. Additional surges were recorded in summer 2022, driven by SARS-CoV-2, and during the unusually early RSV epidemic after the lifting of COVID-19 restrictions [23]. It should be noted that the consultation percentage was not specific for influenza infection, and therefore, the ILI proportion may reflect influenza virus, SARS-CoV-2, or other respiratory infections, such as *Mycoplasma pneumoniae* infection, resulting in ILI symptoms and subsequent GP consultation.

Laboratory analysis showed distinct patterns for several respiratory viruses. Swab testing showed that the surges in SARS-CoV-2 infections may be due to a shift in variant, e.g. Omicron to recombinant, waning immunity and/or shifts in the vaccination programme. Endemic coronaviruses circulated at similar levels in all age groups, whereas SARS-CoV-2 detection remained more common in adults aged 65 years and above. A previous study of young children showed that endemic coronavirus infection can lead to strong immunity towards the same virus but not against other coronaviruses, though the longevity of this immunity is not yet fully understood [24]. Taken together, these findings imply that SARS-CoV-2 will gradually adopt an endemic coronavirus-like pattern of near-continuous circulation punctuated by variant-driven peaks.

In contrast, RSV has shown unusually early epidemics that may be explained by an immunity debt accumulated during the COVID-19 pandemic [23]. Surveillance also detected that parainfluenza virus detection typically aligned with an epidemic immediately following the peak of the influenza epidemic, similar to the sentinel surveillance in Norway [25].

Like other European countries [22], Denmark saw a decline in submitted swabs during lockdown periods. In the first lockdown, starting 11 March 2020, the sentinel surveillance system upscaled and offered one of the few tests for SARS-CoV-2 from week 13 2020 until the national testing system known as TestCenter Denmark was established [26]. During this time GPs offered teleconsultations and discouraged in-person consultations of patients experiencing respiratory symptoms. Together with easy access to at-home antigen tests for SARS-CoV-2, this may explain reduced SARS-CoV-2 detection among the sentinel swabs in 2021/22 despite much higher incidences found in other national surveillance systems [27,28].

To our knowledge, the Danish primary care sentinel surveillance is the first to report individual symptom occurrence for each viral infection. Although specific symptoms varied slightly across different virus families, caution is warranted when interpreting these findings in the context of viral infections. The sentinel system inherently selects patients with a specific symptom presentation, which may not fully represent the broader spectrum of each infection. Although few studies specifically investigate symptom presentation during endemic coronavirus infections, existing literature suggests potential variations in symptom profiles across different endemic coronavirus strains [24,29]. 

Sex-differentiated symptoms showed differences in what women and men seeking healthcare reported to their GP, with up to a 15% frequency difference. Some symptoms, like fever, were more frequently reported among men, while women reported headaches more regularly. The symptom checklist was paper based, meaning that the absence of a mark does not inherently indicate the absence of a symptom. Instead, it may reflect uncertainty or simple oversight during submission. A digital version could mitigate this problem and would offer a more complete dataset needed to elucidate differences in symptoms both by viral infection and demographic.

Our study had some limitations. The reported symptoms show that while sudden onset was the only singular symptom required for inclusion in the surveillance, not all patients reported this, and many did not report symptoms fulfilling the ILI case definition. Furthermore, if the symptom is not explicitly defined during the consultation, it may lead to discrepancies in recognition and reporting. Leniency towards the ILI case definition may have allowed patients with less severe illness to be included in the surveillance, possibly accounting for the many negative results or over-representing milder cases. Informal communication with SPs indicated that, while GPs valued the insight into circulating viruses, drop out was primarily because of the time-consuming nature of submission. This further highlights the need to digitise the reporting system to standardise data entry and improve quality. Planned work to digitise the paper-based workflow aims to streamline swab submission and enhance completeness and accuracy of symptom reporting. Integrating the sentinel system with electronic requisition software already familiar to GPs can minimise extra workload and allow for an automated, real-time database for the sentinel system. 

## Conclusions

The Danish sentinel surveillance has shown that the year-round, integrated approach can detect both expected seasonal patterns e.g. influenza-associated winter peaks, and unveil patterns extending into the interim season e.g. SARS-CoV-2 and parainfluenza virus. Data from this system are routinely communicated to policymakers, clinicians and the general public, thereby strengthening awareness and supporting timely, evidence-based public health decisions.

## Data Availability

The individual-level data used in this study are sensitive and cannot be publicly shared.

## Supplementary Data

344000 Supplement
