# Supplementary material for: Primary care sentinel surveillance of influenza-like illness and laboratory detections of respiratory viral infections in Denmark, week 40 2021 to week 39 2023
Source: Euro Surveill. 2025 Oct 9;30(40):2500103. doi: 10.2807/1560-7917.ES.2025.30.40.2500103 (PMC12514434; doi:10.2807/1560-7917.ES.2025.30.40.2500103)
Supplement: Supplement [file 25-00103_BOLT_BOTNEN_Supplement.pdf]

This supplementary material is hosted by Eurosurveillance as supporting information alongside the article "Primary care sentinel surveillance of influenza-like illness and laboratory detections of respiratory viral infections in Denmark, week 40 2021 to week 39 2023" on behalf of the authors who remain responsible for the accuracy and appropriateness of the content. The same standards for ethics, copyright, attributions and permissions as for the article apply. Eurosurveillance is not responsible for the maintenance of any links or email addresses provided therein.

Supplementary text: Description of each virus' circulation in the Danish Sentinel Surveillance System in 2021/22 and 2022/23 seasons

Adenovirus was present in most weeks throughout both seasons but showed sporadic activity during the summer months (figure 3).

Entero-/rhinoviruses were present in all weeks of both seasons (figure 3). Further characterisation was performed on 53 (16,0%) of enteroviruses and 160 (14,5%) of rhinoviruses. Among the 13 types of enteroviruses detected, the most common were EV-D68 (9), CB02 (6) and CB04 (6, data not shown). In total, 59 rhinovirus types were detected, of which the most common were A68 (15, 9.4%), C15 (10, 6.3%), and A59 (7, 4.4%, data not shown).

Influenza virus was largely absent from Denmark from March 2020, when covid-19 restrictions were first implemented, until all restrictions were lifted in February 2022 (21) (figure 3). This influenza season had an unusually sharp increase and quick decline, resulting in a short season lasting only from week 8, 2022 to week 19, 2022 (21). During these 11 weeks, 685 influenza virus detections were found in the sentinel surveillance, dominated by influenza A, which stood for 99% of cases detected, almost exclusively subtype A(H3N2) (99%). Influenza virus was sporadically detected in the sentinel surveillance for the rest of the season. In the 2022/23 season, there was a co-circulation of influenza A (42%, A(H1N1)pmd09: 57%, A(H3N2): 24%, unsubtyped: 19%) and influenza B (58%).

Metapneumovirus peaked during the autumn-winter of 2021/22, but shifted to winter-spring of 2022/23 (figure 3).

Parainfluenza virus circulated year-round, extending beyond week 20 (figure 3). In both seasons, parainfluenza followed after the influenza epidemic. In 2021/22, parainfluenza type 3 accounted for 71.2% (230) of all parainfluenza detected, followed by type 2 (56, 17.3%), type 1 (20, 6.2%), and type 4 (17, 5.3%). In week 38, 2022, numbers increased, dominated by type 1. The influenza epidemic displaced parainfluenza in week 9, 2022, until decreasing in week 15, 2023. Parainfluenza then increased, dominated by type 3, again showing post-influenza season increase. The 2022/23 season showed mixed circulation: type 3 (97, 41.3%), type 1 (60, 25.5%), type 4 (57, 24.3%), and type 2 (21, 8.9%).

RSV saw an unusually early epidemic occurring from week 36, 2022 to week 3, 2023 (figure 3). RSV-A was primarily found at the start of the 2021/22 season and the end of the 2022/23 season. RSV-B comprised the bulk of RSV-positive swabs in the 2022/23 season, although many were not further characterised.

SARS-CoV-2 was absent for the first six weeks of the 2021/22 season sentinel surveillance, but several waves were seen, starting from February 2022 when all social restrictions related to the SARS-CoV-2 pandemic were lifted in Denmark (22). Sequencing results showed the circulation of delta variants in the very early spring, rapidly replaced by omicron variants (figure 3), which infected just under 70% of the

population within weeks, as previously reported (23). In particular, a large summer wave was seen in 2022. The highest occurrence of SARS-CoV-2 during the 2022/23 season was a winter wave from week 40, 2022 to week 5, 2023 (202 positive swabs). There was a spring wave from week 10, 2023 to week 20, 2023 as well as a late summer wave starting in week 34, 2023 continuing through the end of the study period.

Endemic coronaviruses circulated almost continuously throughout both seasons (figure 3). In 2021/22, a prolonged wave was dominated by OC43 (56%). At the start of the season, 25% of detected endemic coronaviruses were 229E, then joined by HKU1 (15%) and NL63 (4%) in the spring of 2022. The 2022/23 season had a larger but shorter epidemic dominated by OC43 (49%) followed by NL63 (34%), HKU1 (11%), and 229E (7%).

Supplemental material

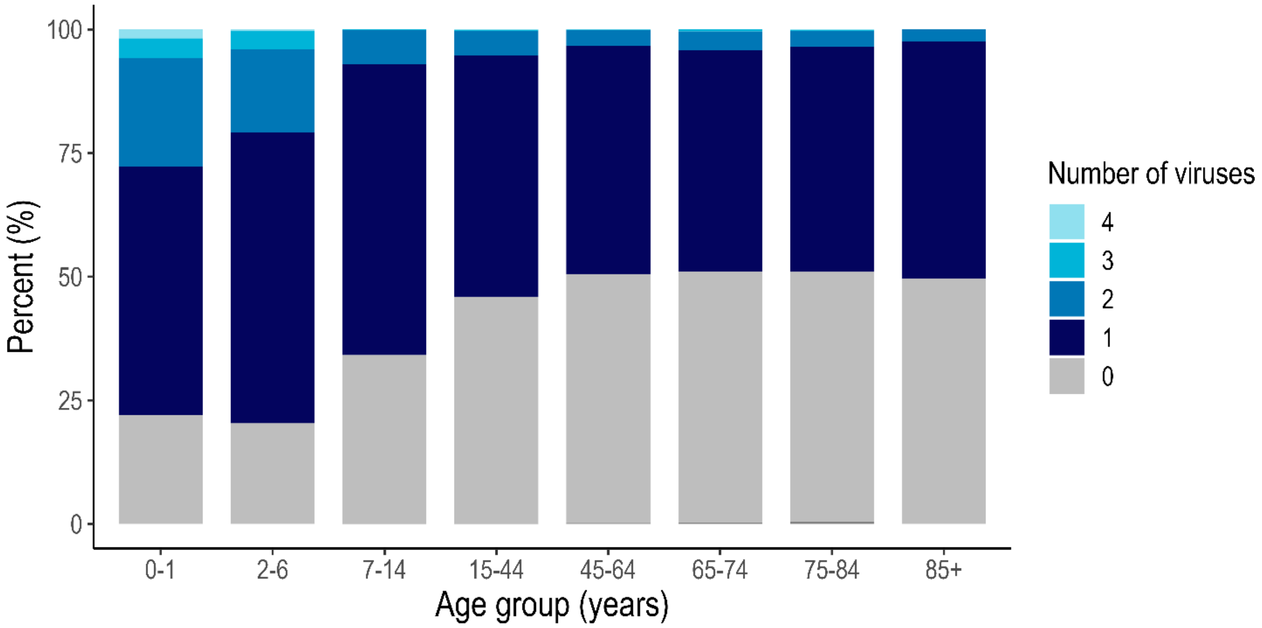

Figure S1: Number of viruses found in each patient swab during the 2021/22 and 2022/23 seasons in Denmark.

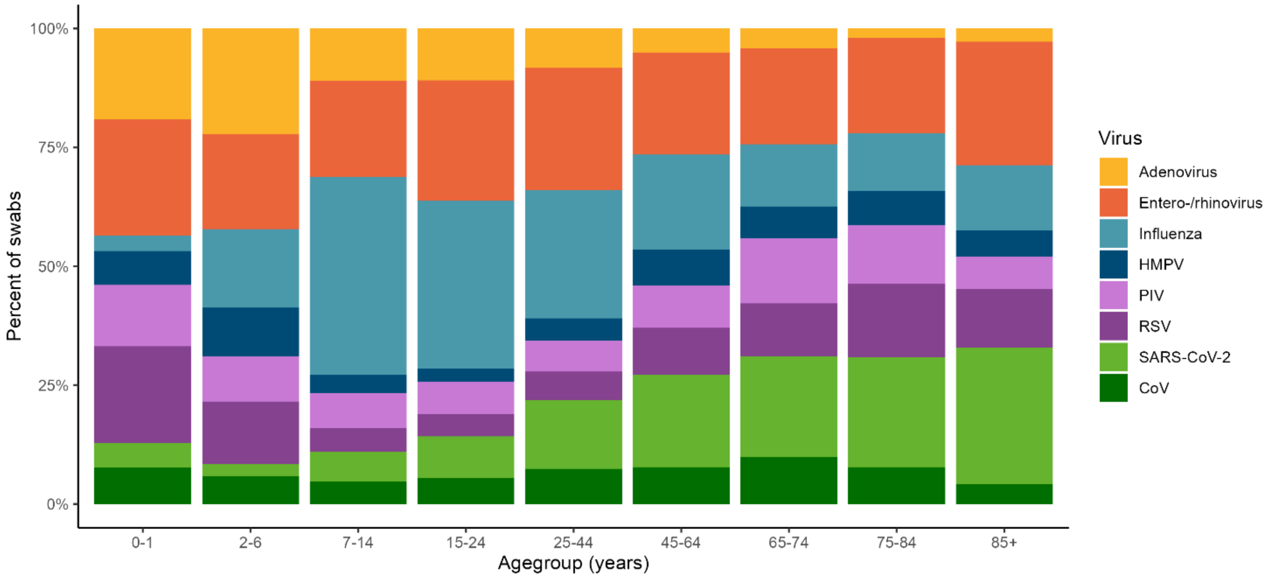

Figure S2: Distribution of viruses per age group, in the two seasons of sentinel surveillance.

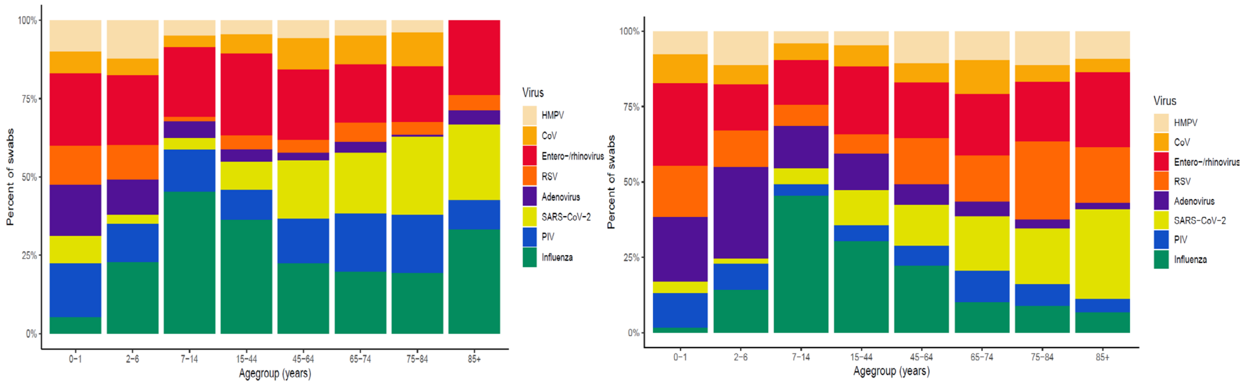

Figure S3. Left: season 2021/22, right: season 2022/23
